# Effects of high-intensity interval training in more or less active mice on biomechanical, biophysical and biochemical bone parameters

**DOI:** 10.1038/s41598-021-85585-9

**Published:** 2021-03-19

**Authors:** Emanuel E. C. Polisel, Wladimir R. Beck, Pedro P. M. Scariot, Taciane M. M. Pejon, Claudio A. Gobatto, Fúlvia B. Manchado-Gobatto

**Affiliations:** 1 Laboratory of Applied Sport Physiology, School of Applied Sciences, University of Campinas, Pedro Zaccaria Street, 1.300, Jardim Santa Luíza, Limeira, SP 13484-350 Brazil; 2 Laboratory of Endocrine Physiology and Physical Exercise, Department of Physiological Sciences, Federal University of São Carlos, São Carlos, SP Brazil

**Keywords:** Biophysical chemistry, Biochemistry, Biophysics, Physiology, Structural biology, Health care, Risk factors

## Abstract

High-intensity interval training (HIIT) is of scientific interest due its role in improving physical fitness, but the effects of HIIT on bone health need be carefully explored. Further, it is necessary to know whether HIIT effects on bone health are dependent on the physical activity levels. This may be experimentally tested since we have built a large cage (LC) that allows animals to move freely, promoting an increase of spontaneous physical activity (SPA) in comparison to a small cage (SC). Thus, we examined the effects of HIIT on biophysical, biomechanical and biochemical parameters of bone tissue of C57BL/6J mice living in cages of two different sizes: small (SC) or large (LC) cages with 1320 cm^2^ and 4800 cm^2^ floor space, respectively. Male mice were subdivided into two groups within each housing type: Control (C) and Trained (T). At the end of the interventions, all mice were euthanized to extract the femur bone for biophysical, biomechanical and biochemical analyses. Based a significant interaction from two-way ANOVA, trained mice kept in large cage (but not for trained mice housed in SC) exhibited a reduction of tenacity and displacement at failure in bone. This suggests that long-term HIIT program, in addition with a more active lifestyle correlates with exerts negative effects on the bone of healthy mice. A caution must also be raised about the excessive adoption of physical training, at least regarding bone tissue. On the other hand, increased calcium was found in femur of mice housed in LC. In line with this, LC-C mice were more active (i.e. SPA) than other groups. This implies that an active lifestyle without long-term high intensity physical training seems to play a role in promoting benefits to bone tissue. Our data provides new insights for treatment of osteo-health related disorders.

## Introduction

High-intensity interval training (HIIT) is broadly defined as repeated bouts of short to long duration exercise completed at an intensity greater than the anaerobic threshold interspersed with recovery periods^1^. It has been applied to different populations as an effective method to increase energy expenditure, improve physical fitness and body composition^2,3^. The main justifications for adherence to this method are the rapid metabolic adaptations promoted and the shorter time allocated to the training session^4,5^. However, even with the increasing number of investigations aimed at analyzing the main physiological and metabolic effects of HIIT in several populations, few studies have shown concern as to following a long-term experimental design applying the high-intensity training^6,7^. Considering that positive or negative adaptations can be achieved when training protocols are carried out for long periods, it is important to conduct studies to assess the chronic effects of this method. This is further strengthened in groups considered to be at high risk, which have problems or physio pathologies that require care when using intense physical exercise.

In this sense, sedentary or obese individuals need caution or prior preparation to undergo HIIT, as they are more sensitive to the consequences of high-intensity exercise. The tissues (systems) that may be affected and require attention include the bones, which are absolutely related to quality of life and sensitive to physical training. Accordingly, studies have observed a positive effect of aerobic exercise on the reduction of bone resorption in healthy young individuals^8^, prevention of osteoporosis and increase in bone mineral density in menopausal women^9–12^, and increase in mineral density in mice^13^. In addition, it has been observed that the muscle contraction is the most important physiological load to be subject on the bones, potentially modifying its biomechanical and biophysical properties as explained through mechanostat theory^14–16^.

Although the literature has developed with regard to the effects of physical exercise on bone health, the actual impact of such intensity on this tissue is still not fully understood. In this sense, it has been observed that high-intensity training can generate significant loss of cortical bone, favoring the appearance of injuries or fractures^17,18^. Additionally, some studies indicate that high-intensity efforts can elevate the C-terminal telopeptide region of collagen type 1 (β-CTX) and the parathyroid hormone (PTH), which are markers of bone resorption, resulting in weakened bone tissue and increasing the risk for fracture, especially in obese individuals^19,20^. Therefore, considering the impact of programs of this nature on different physical and physiological aspects, as well as the need to obtain more benefits than risks, attention should be directed to the effects of HIIT practice on bone tissue in sedentary individuals. Obviously, performing experiments of this nature in humans is not simple.

From this perspective, rather than high-intensity exercise, high-volume exercise seems to contribute positively to bone tissue^13,21^. In order to meet these research needs, our group developed a model capable of promoting different housing space conditions for rodents, enabling clear characterization of the groups according to different levels of spontaneous physical activity (SPA). In this model, animals living in small cages show reduced SPA levels, while rodents living in large cages became more active^22^. We consider this method effective to compare the effects of HIIT on the bone tissue of more or less active animals, according to the housing space to which they are submitted.

Therefore, the main objective of this study is to investigate the effects of long-term HIIT program applied to mice living in small or large cages on biophysical, biomechanical and biochemical parameters of bone tissue. Our main hypothesis is that HIIT will be able to increase stress on bone tissue and, consequently, decrease fracture resistance in this tissue, especially when applied to mice with low SPA level.

## Materials and methods

### Animals

Forty C57BL/6J mice were kept in air-conditioned environment at 22 ± 1 °C, relative humidity between 45 and 55%, noise below 80 dB and photoperiod with a controlled light/dark cycle (lighting from 6:00 h to 18:00 h) throughout the experimental period. All methods were carried out in accordance with relevant guidelines and regulations. All procedures and protocols were approved by an ethical review committee (*Comissão de Ética no Uso de Animais*-CEUA-UNICAMP, protocol number 4953−1/2018).

### Experimental design

After weaning, at 29 days of age, 40 male mice arrived from the central animal care facility were housed in a local animal care facility to environmental adaptation of 6 days. After this, they were initially divided into two groups according to the type of housing: small cage (SC) and large cage (LC), where they were kept for 4 weeks. Subsequently, at 63 days of age, these rodents were again subdivided into two other experimental groups, namely control group (C), which did not perform training in running, and trained group (T), which was submitted to HIIT, totaling 4 groups (SC-C, SC-T, LC-C, and LC-T, n = 10 *per* group). These two types of housing (SC and LC) were considered as environments for the development of less and more active animals, respectively. Thereafter, the interventions were conducted for 13 weeks, including application of the critical velocity (CV) protocol (at the beginning and middle period) to individualize the intensity of HIIT program of 10 weeks. Additionally, SPA was measured at the first, fifth and tenth weeks of training. At the end of the study, all mice were euthanized to extract the femur bone for biophysical, biomechanical and biochemical analyses.

### Housing conditions

The SC group mice were kept in polypropylene boxes with dimensions of 40 × 33 × 16 cm (length, width and height, respectively). For the LC groups, the animals were kept in boxes with dimensions of 80 × 60 × 33.3 cm. The floor areas for the SC and LC groups were 1320 cm^2^ and 4800 cm^2^, respectively. In both housing conditions, the 63-day-old mice were kept at a density of 10 animals per cage (132 and 480 cm^2^ for each animal for SC and LC, respectively).

### Critical velocity protocol

Critical velocity protocol was used to determine critical velocity (CV), which may be taken as a measure of aerobic capacity^22–25^*.* Further, CV demarcates the lower bound of the severe intensity exercise domain, standing slightly above the anaerobic threshold intensity^26^. CV protocol is based on the well-established mathematical relationship between exercise intensity and time to exhaustion (tlim) for different intensities. Animals were submitted to four randomized runs (exhaustive efforts) of running in a calibrated treadmill. CV protocol was done in four consecutive days. Exercise intensities (ranging between 18 and 27 m.min^−1^) were individually selected so that the tlim to was not more than 15 min and not less than 1 min following protocol recommendations^23^. The tlim was considered when the mice were unable to run properly on the treadmill, despite encouragement given by the researcher (without electrical stimulus). CV was calculated from the slope (angular coefficient) of the linear regression fit between distance vs tlim (from each exhaustive effort).

### High-intensity interval training (HIIT)

**Figure 1:**
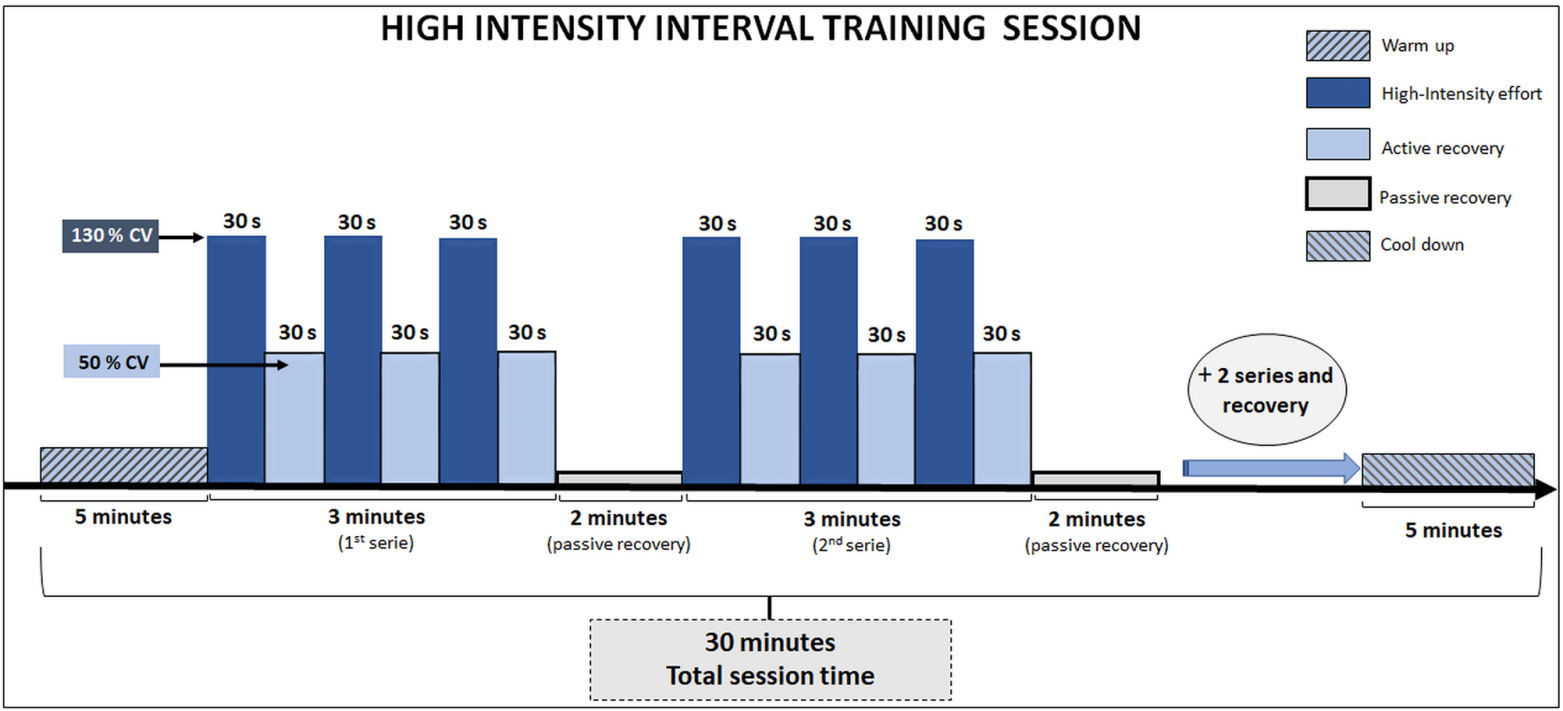
Illustration of a HIIT session including all information about intensity, volume, series, and recovery. *CV* : critical velocity.

Initially, the rodents were adapted to running on a treadmill for 3 days before the start of physical assessment and HIIT procedures. In this adaptation period, the mice ran at an intensity between 8 and 17 m.min^−1^, with exercise volume ranging between 10 and 15 min. After CV determination, HIIT was started. In each session (exemplified in Fig. 1), the mice of the trained groups performed a warm-up at 8 m.min^−1^, for 5 min. The main portion of the training session consisted of four series of three minutes. Each series included three running efforts (30 s) at intensity equivalent to 130% of individual CV (about 28 m.min^−1^), interspersed with active recovery (30 s at 50% of individual CV, about 11 m.min^−1^). A two-minute passive rest was performed at the end of each series. After the main portion, the rodents were submitted to a cool-down period consisting of a 5-min walk at 8 m.min^−1^. Therefore, the total exercise session time was equivalent to 30 min. The training was carried out 5 times a week and HIIT was applied between 7:00 h and 11:00 h. Non-trained mice were also transported to the same location where the trained mice were manipulated. This type of manipulation for control groups has been used to minimize the influence of environmental stress, without promoting, however, physiological adaptations confusing the effects of HIIT.

### Measurement of spontaneous physical activity (SPA)

SPA was measured by the gravimetric method^27^, according to the activities produced by the animals that generated a force on the platform, recorded by load cells capable of identifying them as weight changes on the structure. The gravimetric principle has been used successfully on many studies involving animal models^22,28–32^. In each housing condition (SC and LC), we used three load cells of similar design (MKPM, MK Controle e Instrumentação, BR)^22^. The three load cells were arranged into a triangular layout to detect the variation in force on the metallic platform. The instruments used to amplify and condition the signals were MKTC5−10, (Controle e Instrumentação, BR) and NI-USB 6008 (National Instruments, USA) and SC-2345-SCC (National Instruments, USA). The signals were transmitted to digital acquisition software (LabVIEW Signal Express National Instruments, USA). Although the validity of the gravimetric method has been already demonstrated^27^, we performed calibrations for checking the linearity and stability of the system. The gravimetric method was calibrated by positioning known weights on top of each load cell. Signals (in volts) were converted to grams units by using the linear regression equations (all calibrations showing R^2^ ≥ 0.99) obtained from calibrations plots. We obtained three regression equations since our apparatus was supported by three load cells.

With regard to practical procedures, the SPA recording was started at 12:00 h and continued until 6:00 h. Therefore, SPA recordings were performed for 18 continuous hours (12 h of dark period and 6 h of light period). Mice had SPA evaluated at the beginning (1st week), in the middle (5th week) and at the end of experiment (10th week), being studied therefore 7 days in each of these weeks. Considering that animal handling (HIIT, cage cleaning) was performed between 6:00 h and 12:00 h, this time period was excluded from the analysis. SPA values from 12:00 h to 18:00 h (lamp on) were used to calculate the rodents’ mean activity in the light period, while SPA values from 18:00 h to 6:00 h (lamp off), for the dark period. It was intended to obtain SPA data from the entire dark period since mice display well-defined nocturnal activity^33^. In this regard, disturbance of natural behavior of mice (animal handling, human presence) during SPA recordings was avoided. We measured SPA on a per cage basis since cage isolation has been discouraged^34,35^. It is known that changes in housing density or cage isolation may generate social instability^34–38^. As single housing in order to obtain individual values could be deleterious even if kept as short as possible, the mice were kept collectively to preserve a harmonious social environment.

SPA was obtained according to a mathematical strategy proposed by Biesiadecki et al.^27^, as detailed in following lines. Signals were processed by using Matlab software. A butterworth filter (4-order, low-pass) with a cutoff frequency of 5 Hz was used to remove electrical noise of signals. For SPA calculations, signal variations were obtained by taking the difference between two consecutive signals (*S*). Signals variations values were squared, and the square root was taken for converting all variations to absolute values or *modulus*. This conversion procedure was used in order to eliminate negative values since downward movements (produced by mice) register positive values whereas upward movement register negative values^27^. Finally, SPA was determined by the sum of variations of weight, in absolute values, as is described in the equation below:

$$SPA= \sum_{i=1}^{n}\sqrt{{\left({S}_{i+1}-{S}_{i}\right)}^{2}}$$

The equation above was applied throughout all signals, which were captured at a frequency of 200 Hz. At this acquisition frequency and considering the use of twelve load cells (three cells for each housing type), it was captured about 155 million of registers in an only experiment day (18 continuous hours). After all, signal variations from the three load cells were summed to provide a single value corresponding to the total amount of movements acting on the platform. Thus, SPA of cage was obtained by the sum (resultant) of signal variations recorded by three load cells. Lastly, the SPA value was divided by the sum of the body mass (grams) of all animals in the cage. For example, the SPA values were divided by the mass of all mice of cage 300 g (taken ten mice weighting 30 g, for example).

### Obtaining and processing biological materials

#### Analysis of bone tissue

The animals were euthanized by cervical dislocation followed by decapitation. This process was carried out forty-eight hours after the last session of the CV protocol. After euthanasia, the right femur was collected, kept in saline (NaCl 0.9%) and stored at − 20 °C for subsequent biophysical, biomechanical, biochemical and biometric analyses. The choose for right femur was just conducted according for standardization procedure, assuming that we have no reason to find differences between right or left femur within healthy animals. Before frozen, the bones have all of the minor residues of soft tissue mechanically removed in order to avoid any interference.

#### Biomechanical testing

**Figure 2:**
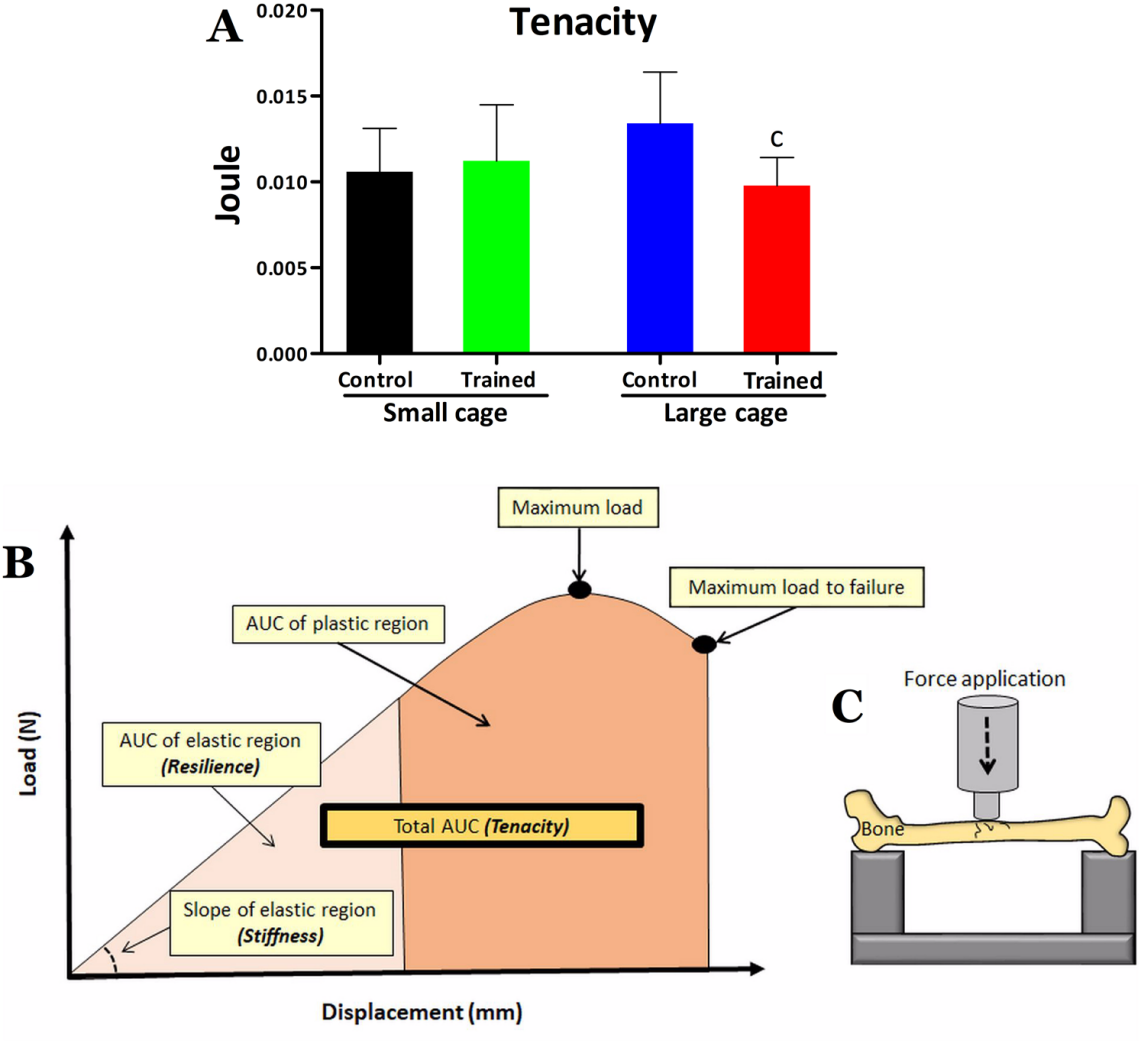
Femoral tenacity for the control and trained groups kept in small cage or large cage in (**A**). Tenacity is regarded as the total area under curve from a typical load *vs* displacement curve of bone (**B**). Additionally, (**B**) illustrates the resilience and stiffness, which are respectively the area under curve (AUC) of elastic region and slope of elastic region. (**C**) Illustrates how the bone was biomechanically tested using Instron Universal Machine. Statistical significance symbol: c different from LC-C (P < 0.05).

The fixture geometry of the bones was assessed and indicated a difference of femur length less than 2 mm among groups, avoiding individual adjustments for the biomechanical tests^39^. The major difference among groups was 0.44 mm (15.56 mm for SC-T and 16 mm for LC-C and LC-T; 2.7 5%). The mean femur diameter was from 0.29 to 0.3 mm for all groups. For biomechanical tests, the bones were initially desiccated for 24 h to remove air from the pores. Subsequently, wet weight (WW) was registered through an electronic analytical scale and immersed weight (IW) was measured attaching the bone through a cooper line to a structure above the same scale and positioning it inside a Becker with water, for later determination of biophysical parameters. As illustrated in panel C of Fig. 2, the bones were then submitted to a three-point bending tests in Instron Universal Machine testing (Model 4444, Instron Corporation, Canton, MA, USA), using the Instron series IX automatic materials tester version 8.09.00 software (S/N 604423C) under standard test conditions. The bones were supported in metaphyseal regions and tested in the same orientation, with the anterior cortex allocated in compression and the posterior cortex in tension. The 1-N preload was applied to prevent the sample from slipping. A constant displacement rate of 5 mm.min^−1^ was applied until bone fracture, with displacement load data collected by the device, with acquisition at 80 Hz. Thus, the biomechanical properties obtained by the graph corresponding to bone deformation load were maximum load (N), displacement at maximum load (mm), resilience (J), tenacity (J), stiffness (N.mm^−1^), maximum load to failure (N), and displacement at failure (mm)^40,41^.

#### Biophysical properties

After biomechanical tests, the bones were exposed to 100 °C for 24 h, for dehydration and dry weight (DW) measurement. To obtain the ash weight (mineral weight: MW), the bones were placed in a muffle furnace, at 800 °C for 24 h. The biophysical properties of the bone were obtained according to the Archimedes principle^42^. The following equations were used: bone volume = WW – IW/water density (cm^3^); bone density = WW/bone volume (g.cm^−3^); bone mineral density = MW/bone volume (g.cm^−3^); bone water percentage ([100 · (WW − DW)/WW)], %); organic material percentage ([100 · (DW − MW)/WW], %) and mineral material bone percentage ([100 · MW/WW], %).

#### Biochemical properties

After all the biomechanical and biophysical procedures, the bone ash obtained was solubilized in hydrochloric acid (2 N), adequately diluted with distilled water. From this solubilized were performed calcium (1:500) and phosphorus (1:1000) dilutions to determine concentrations following the procedures recommended by commercial kits (Labtest, BR), through colorimetric assay. The reading was made using spectrophotometry (SpectraMax i3, Molecular Devices) with wavelength of 650 nm for calcium and 340 nm for phosphorus.

### Statistical analysis

The results were presented as mean and standard deviation (SD). Normality and homogeneity were tested by Shapiro–Wilk and Levene test, respectively. Parametric statistics was adopted, using the analysis of variance (three-factor ANOVA) in order to compare the effects of training (SC-C and LC-C *vs* SC-T and LC-T), housing space (SC-C and SC-T *vs* LC-C and LC-T), and experimental time (10 weeks) on the SPA. Biophysical, biomechanical and biochemical parameters of the femur, collected at the end of the experiment, were analyzed using a two-way ANOVA, to investigate the effects of training (SC-C and LC-C *vs* SC-T and LC-T) and housing space (SC-C and SC-T *vs* LC-C and LC-T), as well interaction. The Newman Keuls post-hoc test was used to indicate differences among groups. The significance level was set at 5%, for all cases.

### Animal welfare statement

The authors confirm that the ethical policies of the journal, as noted on the journal’s author guidelines page, have been adhered to and the appropriate ethical review committee approval has been received. All methods were carried out in accordance with relevant guidelines and regulations. The authors confirm that they have followed national and international standards for the protection of animals used for scientific purposes. All procedures and protocols were approved by an ethical review committee (*Comissão de Ética no Uso de Animais*- CEUA-UNICAMP, protocol number 4953−1/2018).

## Results

### Biophysical, biomechanical and biochemical parameters

Table 1 shows the values for the biophysical parameters of the femur after 13 weeks of experiment for all groups. Post-hoc comparisons showed no difference for any parameter between groups. According to the results of the two-way ANOVA (and training effects), no housing or training effect was detected for all biophysical parameters.

**Table 1:** Bone volume (cm^3^), bone density (g.cm^−3^), bone mineral density (g.cm^−3^), percentage of water, organic and mineral materials from the animals' femur for the control (C) and trained (T) groups kept in small cage (SC) and large cage (LC).

	Small cage		Large cage		Housing space effect		Training effect		Interaction	
	C	T	C	T	P	F	P	F	P	F
Bone volume (cm^3^)	0.09 ± 0.01	0.10 ± 0.01	0.11 ± 0.01	0.10 ± 0.01	0.34	0.89	0.83	0.04	0.21	1.61
Bone density (g.cm^−3^)	1.24 ± 0.03	1.21 ± 0.08	1.24 ± 0.06	1.19 ± 0.08	0.64	0.21	0.05	3.88	0.71	0.13
Bone mineral density (g.cm^−3^)	0.25 ± 0.05	0.22 ± 0.04	0.24 ± 0.05	0.22 ± 0.06	0.74	0.11	0.18	1.84	0.62	0.24
Bone water (%)	61.15 ± 4.19	62.62 ± 2.81	63.69 ± 3.42	61.9 ± 2.56	0.39	0.73	0.88	0.02	0.13	2.31
Organic material (%)	18.23 ± 3.71	18.83 ± 3.63	16.91 ± 2.62	18.6 ± 3.50	0.49	0.47	0.27	1.20	0.59	0.29
Mineral material (%)	20.60 ± 4.07	18.54 ± 3.56	19.38 ± 3.57	19.0 ± 4.26	0.78	0.07	0.34	0.91	0.48	0.49

Data are in mean ± standard deviation (SD). Statistical analysis: We used two-way ANOVA for identifying the effects of housing space and training and housing space and post-hoc Newman Keuls to trace differences among groups.

Regarding biomechanical parameters (Table 2), the post-hoc test traced no difference among groups for maximum load, displacement at maximum load, resilience, stiffness, maximum load to failure, and displacement at failure. However, difference was detected between the LC-T and LC-C groups for the tenacity parameter (Fig. 2), with lower values being observed for the trained animals that lived in a large cage. According to the two-way ANOVA, there was no housing space or training effect for all biomechanical parameters, but there was interaction between the effects for tenacity (F = 5.6; P = 0.02) and for displacement at failure (F = 4.7; P = 0.03). Regarding the biochemical parameters (Table 3), the post-hoc test detected lower calcium values for SC-T compared with the SC-C, LC-C and LC-T groups, as well as SC-C was higher than SC-T and lower than LC-T Group (Table 3). According to the results of the two-way ANOVA, there were housing space effects for calcium values (F = 19.2; P < 0.01; SC < LC) and interaction between the effects (F = 8.9; P < 0.01), but there was no training effect (F = 0.2; P = 0.64) for this variable. Regarding the phosphorus values, there were no differences among groups, with no housing space effect (F = 0.40; P = 0.480), training effect (F = 0.0; P = 1.00) and interaction (F = 0.1; P = 0.69) between them.

**Table 2:** Maximum load (force) (N), displacement at maximum load (mm), resilience (J), tenacity (J), stiffness (N.mm^−1^), displacement at failure (mm) and maximum load (force) to failure (N) of mice femur for the control (C) and trained (T) groups kept in small cage (SC) and large cage (LC).

	Small cage		Large cage		Housing space effect		Training effect		Interaction	
	C	T	C	T	P	F	P	F	P	F
Maximum load (N)	16.23 ± 3.52	15.52 ± 1.85	16.01 ± 2.52	14.86 ± 2.81	0.62	0.23	0.31	1.06	0.80	0.05
Displacement at maximum load (mm)	0.29 ± 0.04	0.31 ± 0.03	0.31 ± 0.08	0.30 ± 0.05	0.84	0.04	0.85	0.03	0.44	0.59
Resilience (J)	0.003 ± 0.001	0.003 ± 0.001	0.003 ± 0.001	0.004 ± 0.002	0.65	0.19	0.36	0.84	0.39	0.74
Tenacity (J)	0.011 ± 0.002	0.011 ± 0.003	0.013 ± 0.003	0.010 ± 0.002^c^	0.43	0.62	0.12	2.4	0.02	5.6
Stiffness (N.mm^−1^)	90.87 ± 21.47	88.89 ± 16.49	84.19 ± 11.22	81.30 ± 17.48	0.20	1.65	0.66	0.19	0.93	0.06
Maximum load to failure (N)	7.51 ± 2.52	8.15 ± 3.04	7.22 ± 1.93	7.42 ± 2.11	0.52	0.42	0.59	0.28	0.78	0.07
Displacement at failure (mm)	1.05 ± 0.19	1.14 ± 0.44	1.46 ± 0.57	1.00 ± 0.18	0.28	1.17	0.16	2.02	0.03	4.72

Data are in mean ± standard deviation (SD). Statistical analysis: We used two-way ANOVA for identifying the effects of housing space and training, and post-hoc Newman Keuls to trace differences among groups. Statistical difference symbol: c- different from LC-C (P ≤ 0.05).

**Table 3:** Calcium and phosphorus values (mg.g^−1^) for the femur of the animals in the control (C) and trained (T) groups kept in small cage (SC) and large cage (LC).

	Small cage		Large cage		Housing space effect		Training effect		Interaction	
	C	T	C	T	P	F	P	F	P	F
Calcium (mg.g^−1^)	227.77 ± 73.23	160.07 ± 37.73^a^	255.07 ± 44.57^b^	304.76 ± 69.81^ab^	< 0.01	19.2	0.64	0.21	< 0.01	8.98
Phosphorus (mg.g^−1^)	110.5 ± 31.70	113.76 ± 21.40	119.46 ± 20	116.27 ± 25.89	0.48	0.49	1.00	0.00	0.69	0.15

Calcium and phosphorus data in mg per gram of bone ash weight are in mean ± standard deviation (SD). Statistical analysis: Two-way Anova was used for identifying the effects of housing space and training, and Post-hoc Newman Keuls was used to trace differences among groups. Statistical significance symbols: a- different from SC-C, b- different from SC-T.

### Spontaneous physical activity

**Figure 3:**
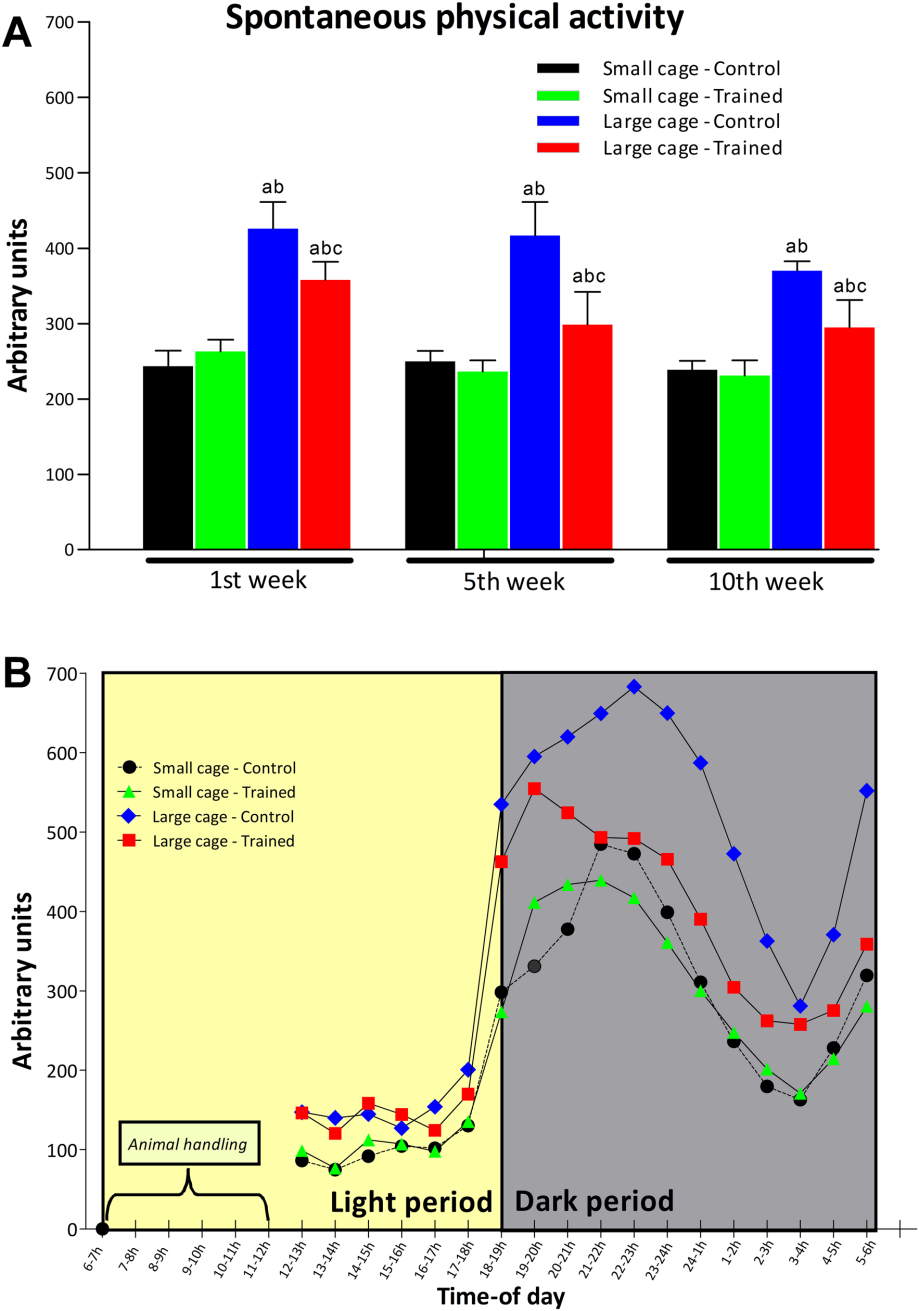
(**A**) shows weekly average SPA in the first, fifth and tenth weeks for the control (C) and trained (T) groups kept in small cage (SC) and large cage (LC). A time-of-day variation of SPA is exhibited in (**B**) demonstrating the robustness and sensibility of gravimetric method. This analysis considers all SPA records obtained in first, fifth and tenth weeks (N = 21 for each hour-time interval from 12:00 to 6:00 h). Animal handling (between 6:00 and 12:00 h) includes HIIT, cage cleaning and weighting control. SPA was measured on a per cage basis (10 mice per cage). Statistical significance symbols: a—different from SC-C, b—different from SC-T and c—different from LC-C within the same experimental week (P < 0.05).

Figure 3 shows the mean weekly SPA in the first, fifth and tenth weeks of training. The post-hoc comparisons indicated that SPA was higher for the LC-C group than the SC-C, SC-T and LC-T groups in the initial, middle and final weeks of training. The LC-T group shows higher SPA than the SC-C and SC-T groups also in the first, fifth and tenth weeks of training. No difference was detected between SC-C and SC-T during the three times of the experiment. According to the results of the three-way ANOVA, there was significant training effect on SPA (F = 55.1; P < 0.01; C > T), showing that the trained groups (SC-T and LC-T groups) exhibited less SPA than the control groups (SC-C and LC-C groups). In addition, housing space had a significant effect (F = 392.5; P < 0.01; LC > SC) on SPA. Finally, experimental time has a significant effect on SPA (F = 14.5; P < 0.01) showing a reduction in SPA over time. Still, interaction was found between training and housing space effects (F = 53.7; P < 0.01) for SPA, as well as interaction between training and experimental week (F = 4.1; P < 0.05), and between housing space and experimental time (F = 4.0; P < 0.05).

## Discussion

Our results show that 10-weeks of HIIT applied 5 times per week can influence bone tissue, as well as change of femoral bone calcium concentrations in mice. On the other hand, in an even more pronounced way, living in a large cage and, consequently, increasing SPA generated more benefits to bone tissue. The most interesting finding in the study showed high tenacity value for mice living in large cage, but the association of this active lifestyle with HIIT resulted in reduced bone resistance, negatively impacting tenacity for the trained group in LC when compared with the control group living in the same conditions.

In relation to the other biomechanical parameters, there was only one interaction between the training and housing space effects for displacement at failure, and trained animals from the large cage group (LC-T) indicated a reduction in this parameter. Regarding the biochemical analyses, the SC-T group showed reduced calcium values compared with the other three groups, indicating that the reduced space, in addition to contributing to the rodents being less active, may be unfavorable for the mice’s recovery after intense exercise as in HIIT. Still, at least for the mice models without physiopathologies, the 10 weeks of HIIT were not able to raise the calcium values for the trained groups. Such found is in line with some authors^18–20^, who showed that high intensity training can lead to negative consequences, as weakened bone tissue and increased risk of fracture. Despite calcium bone concentration is not the full or main responsible for bone health and fracture prevention^43^, the more active lifestyle provided by the large housing space contributed to increase calcium values. This interesting result reinforces the positive effects of a more active lifestyle, in this case enabled by a larger, more attractive space that encourages mobility and the performance of non-forced activities. According with our results, at least in our experimental design, higher levels of SPA (similar activity to low-intensity and high-volume exercise) would provide more benefits compared with HIIT. LC-C group presented higher levels of daily SPA compared with the SC-C and SC-T groups, increased the level of calcium in the femur and increased the tenacity in that bone. In contrast, the tenacity data between the SC-C and SC-T groups showed no significant differences, allowing us to think that, despite the high-intensity exercise, the reduction in SPA ends up being a negative dominant effect. Since bone is a highly modulated tissue in relation to the imposed mechanical signal^44,45^, inducing a more sedentary lifestyle probably resulted in this finding.

The main issue raised in the present study refers to the effects of HIIT applied over the long-term on the bone tissue of more or less active organisms. What would be most beneficial for bone tissue, intensity or volume? Or, would HIIT or large cage (stimulating SPA) be more suitable? According to our findings, the second options seem more positive in increasing bone resistance. This interpretation is based on the lower tenacity observed for the most active group (LC), submitted to HIIT, which, in addition to not increasing, caused the reduction in bone resistance in mice. In this sense, although the mechanism by which different exercise intensities regulate bone remodeling remains uncertain^46^, studies in humans have observed a negative effect of exhausting and strenuous exercise for bone resorption and stress fractures among military recruits and elite athletes^17,47^, which corroborates our findings, at least when tenacity is analyzed. Accumulated micro-damage generated by HIIT could be an explanation for a found lower tenacity, however, our study did not measure it. Future studies could be focused in this way.

With regard to the benefits of daily physical activity on bone tissue, our results indicate that quitting a sedentary lifestyle and raising the level of daily physical activity (represented here by SPA) is an important factor to be considered, since the mechanical load has great influence on the process of bone formation, strength and resistance^48,49^. In addition, we emphasize the importance of a long period of time to find greater adaptations of biomechanical parameters, since the same study with a longer duration also showed resistance as to significant deformations in these parameters^50^. Although in our study we considered the effects of HIIT and of exposure to space for several weeks, for bone tissue this time may not have been enough to change biomechanical parameters, so other effects could probably be observed in a further prolonged investigation. Similarly, the absence of difference in biophysical parameters were found in other rodents studies^40,50–52^, especially in healthy animals, without a deleterious effect already established by previous disease.

An interesting aspect to be highlighted is the high level of SPA promoted by the large housing space. As initially expected, the LC contributed to an active routine for the animals, so they showed a high volume of physical activity throughout the day compared to that observed in HIIT groups. In this sense, in the case of mice, daily activities such as restlessness, fidgeting, posture maintenance, foraging, perching and walking are included in the scope of SPA^53^, and together they can be analogous to low-intensity and long-duration aerobic exercise, since this activity is able to stimulate mitochondrial activity^54^, which is directly related to aerobic capacity.

Regarding the biochemical parameters, there was an effect of housing space, so that an increase in femoral bone calcium values was observed for mice kept in LC compared with mice living in SC. It is known that bone tension generated by impact with the ground and the stimulus of muscle contraction are relevant factors in stimulating bone calcium absorption^55^, a phenomenon afforded by higher SPA. At this point, it is appropriate to make some comments with regard to the types of activities included within the scope of SPA, which can be analogous to exercise of low intensity (could be understood as activities performed with less velocity or ground reaction forces) but that could be executed for a long time (offering a prolonged stimulus on bones despite of low mechanical load). It must be remembered that SPA for rodents is not only associated to movements associated with displacement (i.e. ambulation), but also includes activities without horizontal displacement^53^. Even muscle contractions (within SPA), exerting pressure on bone, may also contribute to bone health^14^. Therefore, LC allowed positive adaptations for calcium, while SC apparently had a negative impact on bone calcium concentration, as supported by a significant housing effect^56,57^. Additionally, the long-term HIIT program caused a reduction in bone calcium values in mice kept in a small cage (SC-T), but increased it for mice kept in a large cage (LC-T), as shown by significant interaction. This reveals that HIIT treatment has opposite effects on femoral bone calcium concentration depending on whether the mice are housed in large or small cages. Finally, the sedentary lifestyle provided by the small cage should be highlighted, since it has already been found that physical inactivity increases the risk for hip and proximal femur fractures in middle-aged or elderly individuals^58^, as this condition weakens bone and muscle tissue. However, we must consider that despite the constant activity of osteoclastic and osteoblastic cells, the remodeling process is slow, which leads to some limitations as to finding severe differences in studies^59^. In the case of our model, although with 10 weeks of intervention, we studied a model of mice still considered young adults^60,61^ and not affected by physiopathologies such as obesity, for example. Thus, the sedentary lifestyle in this period possibly has not yet been extreme, to the extent of reproducing the findings reported by other authors^58,62,63^, with more pronounced biophysical changes. However, even if they are not yet elderly, the level of physical activity raised by the large space model employed here has already promoted more positive results when compared with HIIT.

Despite well conducted, our study is not out of criticism. For example, we have used a proper procedure for biomechanical assessment, but not gold standard method for biophysical analysis (dual-energy X-ray absorptiometry—DXA) and for fracture prediction (the high-resolution peripheral quantitative computed tomography—HR-pQCT) like suggested recently by Mikolajewicz et al.^64^. Nevertheless, as shown by Tie et al.^65^, the Archimedes ’Principle is an ancient and valid method for this purpose, and it have been used a lot nowadays^66,67^, but not always capturing small changes in bone size at specific locations. A final point to be discussed is that the present results were obtained with mice trained at light period, and therefore caution must be taken when interpreting and generalizing our findings to humans. In fact, our option was registering the SPA data from the entire dark period. Studies conducted by our group^22,29^ and others^33^ have consistently found that mice display well defined nocturnal activity, indicating that acrophase of SPA occurs in dark period. For that reasons, we conducted the measure the “pure” SPA during dark period without human's influence on rodents.

In summary, our findings suggest that, in the long-term, the high level of daily spontaneous physical activity was more effective in generating benefits to bone tissue than the high-intensity interval training program. Still, this intense training model combined with the more active lifestyle produced negative effects on the bone tenacity of healthy mice, which suggests caution in the excessive adoption of physical training, at least regarding bone tissue.
